# ULK2 is essential for degradation of ubiquitinated protein aggregates and homeostasis in skeletal muscle

**DOI:** 10.1096/fj.201900766R

**Published:** 2019-08-09

**Authors:** Jordan D. Fuqua, Caleb P. Mere, Ana Kronemberger, Jay Blomme, Dam Bae, Kristen D. Turner, Matthew P. Harris, Estevão Scudese, Mitchell Edwards, Scott M. Ebert, Luís G. O. de Sousa, Sue C. Bodine, Ling Yang, Christopher M. Adams, Vitor A. Lira

**Affiliations:** * Department of Health and Human Physiology, The University of Iowa, Iowa City, Iowa, USA;; † Nursing and Biosciences, Federal University of the State of Rio de Janeiro, Rio de Janeiro, Brazil;; ‡ Department of Molecular Physiology and Biophysics, The University of Iowa, Iowa City, Iowa, USA;; § Department of Internal Medicine, The University of Iowa, Iowa City, Iowa, USA;; ¶ Fraternal Order of Eagles Diabetes Research Center, The University of Iowa, Iowa City, Iowa, USA;; ‖ Department of Anatomy and Cell Biology, The University of Iowa, Iowa City, Iowa, USA;; # Pappajohn Biomedical Institute, The University of Iowa, Iowa City, Iowa, USA;; ** Obesity Research and Education Initiative, The University of Iowa, Iowa City, Iowa, USA;; †† François M. Abboud Cardiovascular Research Center, The University of Iowa, Iowa City, Iowa, USA

**Keywords:** ULK1, p62, NBR1, proteostasis, autophagy, aggrephagy

## Abstract

Basal protein turnover, which largely relies on the degradation of ubiquitinated substrates, is instrumental for maintenance of muscle mass and function. However, the regulation of ubiquitinated protein degradation in healthy, nonatrophying skeletal muscle is still evolving, and potential tissue-specific modulators remain unknown. Using an unbiased expression analysis of 34 putative autophagy genes across mouse tissues, we identified unc-51 like autophagy activating kinase (*Ulk*)*2*, a homolog of the yeast autophagy related protein 1, as particularly enriched in skeletal muscle. Subsequent experiments revealed accumulations of insoluble ubiquitinated protein aggregates associated with the adaptors sequestosome 1 (SQSTM1, also known as p62) and next to breast cancer type 1 susceptibility protein gene 1 protein (NBR1) in adult muscles with ULK2 deficiency. ULK2 deficiency also led to impaired muscle force and caused myofiber atrophy and degeneration. These features were not observed in muscles with deficiency of the ULK2 paralog, ULK1. Furthermore, short-term ULK2 deficiency did not impair autophagy initiation, autophagosome to lysosome fusion, or protease activities of the lysosome and proteasome. Altogether, our results indicate that skeletal muscle ULK2 has a unique role in basal selective protein degradation by stimulating the recognition and proteolytic sequestration of insoluble ubiquitinated protein aggregates associated with p62 and NBR1. These findings have potential implications for conditions of poor protein homeostasis in muscles as observed in several myopathies and aging.—Fuqua, J. D., Mere, C. P., Kronemberger, A., Blomme, J., Bae, D., Turner, K. D., Harris, M. P., Scudese, E., Edwards, M., Ebert, S. M., de Sousa, L. G. O., Bodine, S. C., Yang, L., Adams, C. M., Lira, V. A. ULK2 is essential for degradation of ubiquitinated protein aggregates and homeostasis in skeletal muscle.

Skeletal muscle mass and contractile function are strong independent predictors of positive prognosis and rate of recovery in patients at intensive care units (1). Maintenance of skeletal muscle mass and function also preserves independence and reduces mortality in the elderly (2–5). The physiologic contributions of skeletal muscle to whole-body homeostasis are several. Besides its role in voluntary movement and in supporting respiration *via* diaphragm action, skeletal muscle helps preserve bone mass (6), clears most blood glucose in response to insulin (7, 8), and is the major source of amino acids for hepatic gluconeogenesis under conditions of energy stress such as in fasting or starvation (9). Therefore, a better understanding of the mechanisms involved in the maintenance of skeletal muscle mass and function is of paramount clinical importance.

Appropriate protein degradation and turnover is essential for maintaining skeletal muscle health throughout life. Because of its long-living nature and the expression of a high number of cytoskeletal proteins prone to misfolding and aggregation, skeletal muscle fibers are under a constant proteotoxic challenge (10, 11). Indeed, skeletal muscle is directly compromised by proteotoxic gene mutations such as in amyotrophic lateral sclerosis (12) and Huntington’s disease (13), or by defects in proteolysis such as in Danon disease (14), Pompe disease (15), and collagen VI muscular dystrophy (16). In addition, deficient protein degradation is a feature of age-related muscle weakness (17, 18). To this matter, skeletal muscle protein degradation is executed by several proteolytic systems, including calpains (19), caspases (20), the proteasomal system (21), and the autophagy-lysosomal system or macroautophagy (22). Importantly, the selective removal of cellular proteins in muscle largely relies on their ubiquitination followed by proteasomal or autophagy-lysosomal degradation (23–25), the latter hereafter referred to as autophagy. However, the molecular coordination of ubiquitinated protein degradation in skeletal muscle remains insufficiently understood.

Autophagy is a multistep process by which the largest variety of ubiquitinated cellular substrates, including long-lived proteins, insoluble protein aggregates, and organelles, are degraded (26). Still, the potential skeletal muscle–specific regulators of the autophagy pathway that may affect ubiquitinated protein degradation remain unknown. In the current study, we sought to identify new skeletal muscle–specific regulators of ubiquitinated protein degradation among putative or established autophagy genes. With the perspective that the skeletal muscle proteome requires tailored degradation and turnover, we hypothesized that essential factors modulating protein turnover would be enriched in skeletal muscle under normal, nonatrophying conditions. Our results revealed unc-51 like autophagy activating kinase (ULK)2 to be an essential protein for degradation of ubiquitinated proteins and homeostasis in skeletal muscle. Furthermore, our findings reveal that ULK2 does not directly regulate autophagy in skeletal muscle, thereby having a distinct function in relation to its better-studied paralog ULK1. These findings may have potential therapeutic implications for conditions of poor protein homeostasis in muscle such as in several myopathies and aging.

## MATERIALS AND METHODS

### Bioinformatic analysis of mRNA transcripts encoding proteins with established or putative roles in mammalian autophagy

An initial list of 36 mRNA transcripts encoding proteins with established or putative function in autophagosome nucleation and elongation was established based on previous studies (26–29). Their expression across tissues was determined by querying a mouse tissue profiling array (BioGPS data set: Gene Atlas MOE430, gcrma) (30). Data for microtubule associated protein 1 light chain 3 (Map1lc3) c [also known as light chain 3 (Lc3) c] and chromatin assembly factor 1, subunit A genes were not available, and the final analysis was then performed on 34 mRNA transcripts. First, the degree of skeletal muscle enrichment for each transcript was determined by the ratio between its expression in skeletal muscle and its median expression across all tissues (*i.e.*, 91 tissues and cell lines) using specific gene probes. With the exception of 8 mRNA transcripts, for which data from just 1 probe were available, this ratio was determined from the mean ratios obtained from the 2 probes per mRNA transcript whose expression correlated the highest across tissues. Next, the degree of skeletal muscle–specific expression of the 34 mRNA transcripts was further established by identifying which transcripts were at least moderately correlated (*r* > 0.5) with the tissue-wide expression of 3 skeletal muscle–specific mRNA transcripts (*i.e.*, actinin 3, calsequestrin 1, and myozenin 1). These mRNA transcripts were selected because they encode proteins with distinct annotated molecular functions specific to skeletal muscle, and their expressions are not known to vary across skeletal muscle fiber types and are very closely correlated across tissues (*r* ≥ 0.95). Because only *Ulk2* correlated with these 3 skeletal muscle–specific mRNA transcripts, we then performed a functional annotation clustering analysis on mRNA transcripts whose expression was highly correlated with *Ulk2* across tissues (*r* ≥ 0.8; *n* = 129 genes), using the Database for Annotation, Visualization and Integrated Discovery (DAVID) (31, 32) with medium stringency settings (*https://david.ncifcrf.gov/*). From those, 123 genes were recognized by DAVID and were therefore included in the analysis to reveal potential cellular and molecular features closely associated with *Ulk2* expression.

### Animal models

Male C57BL/6J mice (000664, *n* = 96) were obtained from The Jackson Laboratory (Bar Harbor, ME, USA) and studied at 7–10 wk of age. Mice were housed in Medical Laboratories (University of Iowa) in temperature-controlled (21°C) quarters with a 12:12-h light/dark cycle and free access to water and chow. Electroporation of plasmids to tibialis anterior (TA) muscle was performed as previously described by Ebert *et al*. (33). Essentially, muscles were injected with a 0.4 mg/ml hyaluronidase solution in saline, and 2 h later, muscle TAs were injected with appropriate plasmids in saline solution. DNA plasmids (20 μg) encoding either *Ulk1* or *Ulk2* pre-microRNAs (miRs) were injected into the TA muscle of 1 leg, and a control miR plasmid was injected into the contralateral leg of the same mouse. Immediately following injection, the legs were exposed to 10 electric pulses (20 ms) of 175 V/cm (with 480-ms intervals between pulses) using an ECM-830 electroporator (BTX, Holliston, MA, USA) to ensure incorporation of the plasmids into myofibers. Muscles were harvested 1 or 4 wk afterwards in the morning either at basal conditions (*i.e.*, with mice having normal access to food) or after 24 or 48 h of starvation. Electroporated TA muscles were used for all experiments except for mRNA copy numbers of *Ulk2* and *Ulk1*, in which nonelectroporated plantaris muscles were used instead. Plantaris muscles were used because: *1*) all TA muscles were electroporated with a plasmid-miR construct, which could potentially confound the results; *2*) the plantaris muscle, similar to TA, has a mixed fiber type composition in mice; and *3*) we wanted to minimize the number of experimental animals used. Tissue samples were harvested after mice were euthanized with CO_2_ followed by cervical dislocation. All animal protocols were approved by the Institutional Animal Care and Use Committee of the University of Iowa.

### Plasmids

miR plasmids were generated as previously described by Ebert *et al*. (33). Essentially, p-miR-*Ulk2*, p-miR-*Ulk2* #2, p-miR-*Ulk1*, and p-miR-*Ulk1* #2 were generated by ligating Mmi541282, Mmi541280, Mmi525866, and Mmi525867 oligonucleotide duplexes (Thermo Fisher Scientific, Waltham, MA, USA), respectively, into the pcDNA6.2-GW/EmGFP-miR plasmid (Thermo Fisher Scientific), which contains a cytomegalovirus promoter driving the expression of engineered pre-miRs and emerald green fluorescent protein (EmGFP). p-miR-Control encodes a nontargeting pre-miR hairpin sequence (miR-neg control; Thermo Fisher Scientific) in the pcDNA6.2-GW/EmGFP-miR plasmid. Specificity of miRs targeting *Ulk2* or *Ulk1* was experimentally confirmed by 2 observations. First, we observed efficient reductions in respective mRNA and protein levels by miRs targeting each *Ulk*). To this matter, 2 commercially available antibodies (NBP1-33136; Novus Biologicals, Centennial CO, USA and HPA 009027; MilliporeSigma, Burlington, MA, USA) failed to consistently detect changes in endogenous ULK2 protein levels despite clear reductions in *Ulk2* mRNA in muscles transfected with p-miR-*Ulk2*. To overcome a potential limitation in the sensitivity of these antibodies, we cotransfected (*via* electroporation) a full-length wild-type *Ulk2* construct containing sequential 3× FLAG and *S*-Tag NH_2_-terminal epitope tags (p-WT-*Ulk2*) with either p-miR-Control or p-miR-*Ulk2* and probed muscle lysates for FLAG. FLAG expression was clearly decreased in muscles cotransfected with p-miR-*Ulk2* but not with p-miR-Control, demonstrating the efficiency of miR-*Ulk2* in reducing ULK2 protein in muscle (Supplemental Fig. S1*A*–*C*). Second, we observed that 2 independent miRs targeting each *Ulk* led to consistent effects on autophagy markers (Supplemental Fig. S3). After confirming the specificity of the independent miR sequences for *Ulk2* and *Ulk1*, the plasmids encoding the miRs yielding the strongest *Ulk2* and *Ulk1* mRNA knockdown were then used for subsequent experiments [*i.e.,* Mmi541282 (p-miR-*Ulk2*) and Mmi525866 (p-miR-*Ulk1*), respectively].

### *In vivo* muscle contractile function

In mouse TA muscles, muscle function was assessed by stimulating the common fibular nerve to induce tetanic and maximal isometric contractions of ankle dorsiflexors once a week for 3 wk starting at 7–8 d following electroporation. In each mouse, ULK-deficient and control muscles were tested separately. Briefly, mice were maintained under isoflurane anesthesia on a warming platform kept at 35°C. The knee of the mouse was secured between pivots, and the foot was placed in and secured to the foot plate attached to a force transducer. A total of 2 needle electrodes (monopolar polytetrafluoroethylene-coated stainless steel) (Chalgren Enterprises, Gilroy, CA, USA) were used for percutaneous stimulation of the common fibular nerve. Tetanic isometric contractions at optimal length were elicited by stimulations of 150 Hz for 300 ms using the 1300A 3-in-1 Whole Animal System (1300A; Aurora Scientific, Aurora, ON, Canada). Data were collected and analyzed to determine muscle torque using the Dynamic Muscle Analysis software (ASI 611A v.5.321; Aurora Scientific). Muscles were harvested ≥4 d after the last functional test.

### Colchicine administration

Colchicine (0.4 mg/kg in sterile dH_2_O), a microtubule polymerization inhibitor used to block fusion of the lysosome to the autophagosome, or vehicle (proportional volume of sterile dH_2_O) was administered intraperitoneally at 24 and 12 h prior to tissue harvest as previously described in refs. 34 and 35.

### RNA extraction and real-time quantitative PCR

Skeletal muscle used for real-time quantitative PCR (qPCR) (10–15 mg) was lysed using 1 ml of Trizol reagent to isolate RNA as previously described by Lira *et al*. (36). Chloroform and 100% isopropanol were used to segregate the RNA from lysates and precipitate the RNA, respectively. Isolated RNA was washed twice using 70% ethanol. RNA concentration was determined using the Nanodrop 2000 Spectrophotometer (Thermo Fisher Scientific). cDNA was made from 1 µg of RNA by using a High Capacity cDNA Reverse Transcriptase Kit (Thermo Fisher Scientific). qPCR was performed with a mixture containing the resulting cDNA, primers, and the Power SYBR Green PCR Master Mix (Thermo Fisher Scientific) following standard procedures carried out in QuantStudio 6 Flex Real-Time PCR System (Thermo Fisher Scientific). The primer sequences used are listed in (Supplemental Table S4). The purity of each amplified product was confirmed by melting curve inspections after amplification. Results were normalized to glyceraldehyde 3-phosphate dehydrogenase mRNA, which did not change with our interventions.

### *Ulk1* and *Ulk2* mRNA copy numbers in skeletal muscle

Copy numbers of *Ulk1* and *Ulk2* mRNAs were determined in wild-type mouse muscles. Standard curve templates using 300,000, 30,000, 3000, 300, and 30 copies of the plasmid DNAs encoding either the wild-type *Ulk1* or *Ulk2* were created. qPCR was then conducted as described above, and RNA copy number was determined using the standard curves for each *Ulk* gene.

### Immunoblot analysis

Skeletal muscle samples were prepared, processed, and analyzed as previously described by Lira *et al.* (36). The following antibodies and respective dilutions were used for immunoblots: sequestosome 1 (SQSTM1) (p62) (P0067, 1:1000), ULK1 (A7481, 1:1000), FLAG (F1804, 1:1000) from MilliporeSigma, ULK2 (HPA009027, 1:500) from MilliporeSigma, next to breast cancer type 1 susceptibility protein gene 1 protein (NBR1) (sc-130380, 1:1000) from Santa Cruz Biotechnology (Dallas, TX, USA), LC3A/B (4108, 1:1000), ubiquitin (3936, 1:1000), cathepsin B (CTSB) (31718, 1:1000), lysosomal associated membrane protein 1 (LAMP1) (9091, 1:1000), autophagy related protein (Atg)13 (13273, 1:1000), Atg14 (5504, 1:1000), phosphorylated (p-)Atg14 (S29) (13155, 1:1000) from Cell Signaling Technology (Danvers, MA, USA), ULK2 (NBP1-33136, 1:500) from Novus Biologicals, p-p62 (S403) (MABC186-I, 1:1000) from MilliporeSigma, p-Atg13 (S318) (600-401-C49S, 1:1000) from Rockland (Limerick, PA, USA), and 20S (ab22673, 1:1000) from Abcam (Cambridge, United Kingdom). Results for protein expression were normalized to Ponceau stain.

### Isolation of insoluble protein aggregates

Protein aggregates were isolated using an adapted protocol (11, 37). Essentially, TA muscles (15 mg) were homogenized on ice in mild detergent buffer containing 1% Triton X-100 pH 7.5 with protease inhibitor cocktail (Complete, 11697498001; Roche, Basel, Switzerland) and phosphatase inhibitor (PhosStop, 04906837001; Roche). Tissue homogenates were transferred to 1.0-ml syringes and passed through a 25-G needle 15–20 times. Equal amounts of protein (500 µg) from each sample were then centrifuged at 17,000 *g* for 10 min at 4°C to obtain the supernatant (soluble fraction), which was transferred to new, chilled 1.5-ml tubes. The remaining pellet samples were solubilized in concentrated detergent buffer containing 2% SDS pH 7.5 (insoluble fraction), and water sonicated for 1 h at 4°C with brief vortex every 15 min. Final supernatant and pellet fractions were mixed with sample loading buffer [204 mM Tris·HCl, pH 6.8, 4% sodium dodecyl sulfate (SDS), 40% glycerol, 80 mM dithiothreitol, 570 mM 2-mercaptoethanol, and 0.035% bromophenol blue] (36), denatured at 95°C for 5 min, and then run on SDS-PAGE gels.

### Histology, fluorescence microscopy, and myofiber diameter

For 1-wk experiments, TAs were fixed in 4% paraformaldehyde for 24 h, run through a sequence of incubations at 4°C with sucrose gradient solutions [*i.e.*,10% (w/v) for 1 h, 20% for 1 h, and 30% overnight], and then embedded in tissue-frozen medium. For 4-wk experiments, TAs were directly embedded in tissue-frozen medium for subsequent immunofluorescence analysis. Serial sections of muscle samples (10 µm) were obtained using a Microm HM505E cryostat (Microm International, Walldorf, Germany). Overall muscle morphology and presence of centralized nuclei were assessed in hematoxylin and eosin (H&E)–stained TA sections. Percentage of centrally nucleated myofibers was calculated by dividing the number of transfected fibers containing ≥1 centrally located nucleus by the total number of transfected fibers. An average of 1500 myofibers were counted per muscle, and quantification was performed using ImageJ software (National Institutes of Health, Bethesda, MD, USA). Green fluorescent protein (GFP) and dystrophin immunofluorescence was performed on sections by first postfixing the sections in precooled 4% paraformaldehyde. Next, muscle sections were permeabilized in 0.1% Triton X-100, blocked with normal goat serum (G-9023; MilliporeSigma) diluted to 5% in PBS (5% normal goat serum), and then incubated overnight with primary antibodies (GFP tag A-11122, 1:250; Thermo Fisher Scientific) and dystrophin [MANDRA1(7A10), 1:250; Developmental Studies Hybridoma Bank, Iowa City, IA, USA] at 4°C. On the following day, samples were incubated for 1 h with secondary antibody in 5% normal goat serum and then covered with ProLong Diamond Antifade Mountant (Thermo Fisher Scientific). Of note, samples were washed 3 times with PBS after each of the steps outlined above. Slides were imaged using an Eclipse Ti-S microscope (Nikon, Tokyo, Japan) and an Olympus BX61 Microscope (Olympus, Tokyo, Japan). Image analysis was performed using ImageJ software. In all experiments, muscle fiber diameter was assessed using the minimal Feret’s diameter (*i.e.*, lesser diameter technique) on a minimum of 300 transfected fibers per muscle sample.

### Chymotrypsin-like and CTSB assays

Frozen powdered muscle was homogenized using 150 µl of ice-cold lysis buffer (250 mM sucrose, 20 mM HEPES, 10 mM KCL, 1 mM EDTA, 1 mM EGTA, 1 mM DTT; pH 7.4) without protease inhibitors. Centrifugation was performed for 10 min at 15,000 *g* at 4°C, after which the pellet was discarded and the supernatant was kept for further analyses. A total of 30 μg of protein were used to assess CTSB activity. Homogenates were incubated in activation buffer (25 mM 2-(N-morpholino)ethanesulfonic acid (MES) hydrate, 5 mM DTT; pH 5.0) containing 50 µM Z-Leu-Arg-7-amino-4-methyl coumarin substrate (ES008; R&D Systems, Minneapolis, MN, USA) in a 96-well black plate, and fluorescence was monitored (380 nm excitation, 460 nm emission) every 45 s for 40 min at 37°C in a Spectramax i3 Microplate Spectrofluorometer (Molecular Devices, San Jose, CA, USA). Proteasome assays were performed as previously described in refs. 38 and 39. For the proteasomal ATP-independent chymotrypsin-like activity (20S), 100 µg of protein was incubated in the dark at 37°C with assay buffer (50 mM Tris-HCL, pH7.5, 5 mM MgCl2, 40 mM KCl, 1 mM DTT, and 0.5 mg/ml bovine serum albumin) with or without the selective proteasome inhibitor Epoxomicin (10 µM, 10007806; Cayman Chemicals, Ann Arbor, MI, USA) and 10 µM substrate (Suc-Leu-Leu-Val-Tyr-7-amino-4-methyl coumarin, BML-P802; Enzo Life Sciences, Farmingdale, NY, USA). To assess ATP-dependent chymotrypsin-like activity (26S), 2 mM ATP was added to assay buffer. Fluorescence was monitored (380 nm excitation, 460 nm emission) every 45 s for 1 h, and enzyme activity was determined as change in fluorescence during the linear phase of the reaction.

### Data analysis and statistics

Muscle function, histology, fluorescence microscopy, and morphologic analyses were performed in a blinded manner. Results are presented as means ± sem. A paired Student’s *t* test was used for all data analyses except for force production over time, in which case a 2-way repeated measures ANOVA was used followed by Sidak’s multiple comparison *post hoc* test when applicable. Values of *P* < 0.05 were considered statistically significant. All statistical tests were performed with Prism v.7 (GraphPad Software, La Jolla, CA, USA).

## RESULTS

### Identification of *Ulk2* as a potential regulator of skeletal muscle protein homeostasis

**Figure 1:**
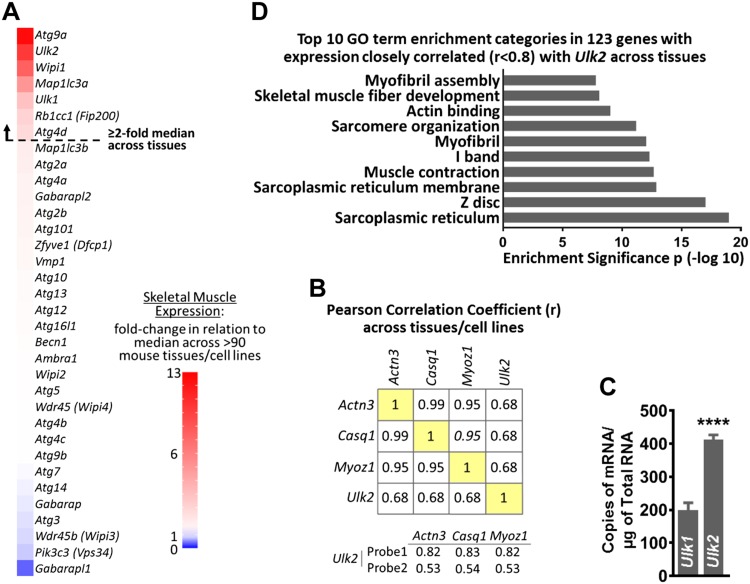
*Ulk2* is enriched in skeletal muscle. BioGPS data set: Gene Atlas MOE430, gcrma (30) was used for *A*, *B*, *D*. *A*) Skeletal muscle expression of 34 putative autophagy genes expressed as fold change over median expression across multiple mouse tissues and cell lines. *B*) Mean correlation between skeletal muscle–specific genes and *Ulk2*. *C*) mRNA copy number of *Ulk2* and *Ulk1* in plantaris skeletal muscle of mice with normal food access (*n* = 9). *D*) DAVID functional annotation clustering analysis showing the top 10 Gene Ontology (GO) categories of genes with the highest expression correlation with *Ulk2* across tissues (*r* ≥ 0.8; *r*2 ≥ 0.64). *Actn3*, actinin 3; *Casq1*, calsequestrin 1; *Myoz1*, myozenin 1. Data are means ± sem. ****P < 0.0001.

To gain new insight into the regulation of protein homeostasis in skeletal muscle, we performed an unbiased analysis of 34 mRNA transcripts that encode proteins with established or putative roles in mammalian autophagy (26–29). We identified 7 mRNAs whose expression was ≥2-fold higher in skeletal muscle relative to ∼90 other mouse tissues and cell lines (BioGPS data set: Gene Atlas MOE430, gcrma) (30) (**Fig. 1*A*** and Supplemental Table S1). These 7 mRNAs included *Ulk1* (a well-characterized serine-threonine protein kinase that mediates autophagy initiation) and its paralog with unknown function in skeletal muscle, *Ulk2*. Next, we examined whether expression of any of the 34 mRNA transcripts correlated with expression of 3 skeletal muscle–specific genes, Actinin 3, calsequestrin 1, and myozenin 1. Interestingly, only *Ulk2* expression strongly correlated with any of these genes (*r* > 0.5) (Fig. 1*B* and Supplemental Table S2). We then complemented this initial *in silico* analysis with qPCR and observed a 2-fold higher copy number of *Ulk2* mRNA *vs.* *Ulk1* mRNA in adult mouse muscle (Fig. 1*C*). Next, we performed functional annotation clustering analysis on the mRNA transcripts whose expression was highly correlated (*r* ≥ 0.8) with *Ulk2* expression across tissues (Supplemental Table S3*A*). These included 123 genes recognized by DAVID (31, 32) and the top 10 Gene Ontology categories identified were all related to muscle (Fig. 1*D* and Supplemental Table S3*B*). Collectively, these data indicate the *Ulk2* gene as a novel potentially important regulator of protein homeostasis in skeletal muscle.

### ULK2 regulates ubiquitinated protein degradation in skeletal muscle

**Figure 2:**
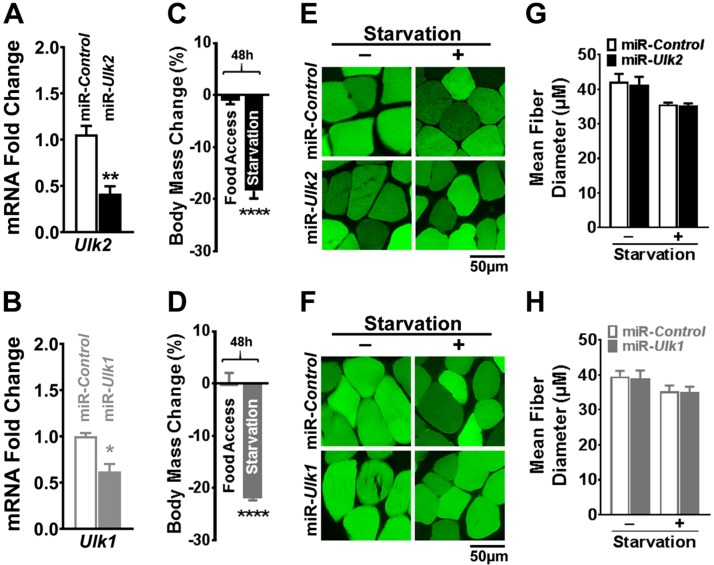
ULK2 and ULK1 are dispensable for starvation-induced atrophy in skeletal muscle. Data were obtained from control and ULK-deficient TA muscles 1 wk after electroporation. *A*) Relative *Ulk2* mRNA in control and ULK2-deficient muscles (*n* = 5). *B*) Relative *Ulk1* mRNA in control and ULK1-deficient muscle (*n* = 4). *C, D*) Percent change in body mass after 48 h of starvation in electroporated mice (*n* = 6–9). *E*) Representative images of transfected fibers (GFP) in control and ULK2-deficient muscles. *F*) Representative images of transfected fibers (GFP) in control and ULK1-deficient muscles. *G*) Quantification of mean fiber diameter of control and ULK2-deficient muscles in mice with normal food access or after 48 h of starvation (*n* = 6–9). *H*) Quantification of mean fiber diameter of control and ULK1-deficient muscles in mice with normal food access or after 48 h of starvation (*n* = 7–8). Data are means ± sem. *P < 0.05, **P < 0.01,****P < 0.0001.

Because ULK2 is considered to be a paralog of ULK1, and ULK1 plays an important role in autophagy, we hypothesized that ULK2 might be required for autophagy in skeletal muscle. To test this hypothesis, we used an electroporation-based technique to transfect mouse TA muscle with a plasmid encoding EmGFP and an artificial miR that specifically reduces ULK2 expression (miR*-Ulk2*). In each mouse, the contralateral muscle received a plasmid encoding EmGFP and a nontargeting artificial miR (miR-Control) that served as an intrasubject negative control, as previously described in refs. 33 and 40 (**Fig. 2*A*** and Supplemental Fig. S1*A*–*C*). To serve as a positive control, a separate cohort of mice was electroporated with a plasmid encoding EmGFP and an artificial miR that specifically reduces ULK1 expression (miR-*Ulk1*). Their contralateral TA was also electroporated with miR-Control (Fig. 2*B* and Supplemental Fig. S1*D, E*). The electroporation process transfects terminally differentiated adult muscle fibers but not satellite cells or connective tissue (41). Muscles were then examined under conditions of normal energy homeostasis or energy stress (*i.e.*, with mice having normal food access or being starved, respectively) because autophagy is known to be stimulated in the latter. Deficiency of either ULK2 or ULK1 did not impact myofiber diameter under normal energy homeostasis or with starvation (Fig. 2*C*–*H* and Supplemental Fig. S2*A, B*), indicating that both ULK2 and ULK1 are dispensable for starvation-induced atrophy in skeletal muscle.

**Figure 3:**
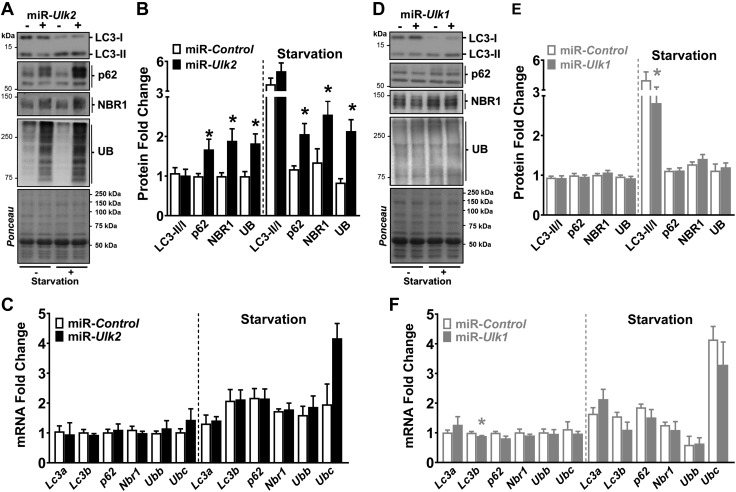
Accumulation of ubiquitinated proteins and autophagy adaptors occur early in ULK2-deficient muscles. Data were obtained from control and ULK-deficient TA muscles 1 wk after electroporation. *A*) Representative immunoblots of LC3 (LC3A/B), adaptor proteins, and ubiquitinated proteins in control and ULK2-deficient muscles of mice with normal food access or after 24 h of starvation. *B*) Quantification of LC3, adaptor proteins, and ubiquitinated proteins (*n* = 5–8). *C*) mRNA levels of genes encoding *Lc3a* and *Lc3b* (*Map1lc3a* and *Map1lcb*, respectively), adaptor proteins *p62* (*Sqstm1*) and *Nbr1*, and ubiquitin [ubiquitin b (*Ubb*) and unibquitin c (*Ubc*)] in control and ULK2-deficient muscles of mice with normal food access (left) and after 24-h starvation (right) (*n* = 5). *D*) Representative immunoblots of LC3 (LC3A/B), adaptor proteins, and ubiquitinated proteins in control and ULK1-deficient muscles of mice with normal food access or after 24 h of starvation. *E*) Quantification of LC3, adaptor proteins, and ubiquitinated proteins (*n* = 6–8). *F*) mRNA levels of the genes indicated in *C* in control and ULK1-deficient muscles of mice with normal food access (left) and after 24 h of starvation (right) (*n* = 4). UB, ubiquitin. Data are means ± sem. *P < 0.05.

ULK2 deficiency had no effect on LC3-I (LC3A/B-I), LC3-II (LC3A/B-II), or the LC3-II:LC3-I ratio in mice with normal food access or starvation. However, ULK2 deficiency generated striking increases of ∼2-fold in ubiquitinated proteins along with the autophagy adaptors p62 (Sqstm1) and NBR1 in mice with normal food access or under starvation (**Fig. 3*A, B*** and Supplemental Fig. S2*C*). Experimentation with a second independent miR targeting the *Ulk2* gene confirmed these findings, eliminating the possibility that these observations were resulting from off-target gene effects (Supplemental Fig. S3*A, B*). Furthermore, the accumulation of these proteolytic substrates was not transcriptionally driven because ULK2 deficiency did not result in comparable, consistent up-regulations of *p62, Nbr1,* or ubiquitin (*i.e.,* ubiquitin b and c) mRNA in these conditions (Fig. 3*C*).

Conversely, ULK1 deficiency led to small increasing trends in both LC3-I and LC3-II when mice had normal food access but caused larger increases in LC3-I *vs.* LC3-II during starvation, resulting in a significantly reduced LC3-II:LC3-I ratio in this condition (Fig. 3*D, E* and Supplemental Fig. S2*D*). This is in line with a role for ULK1 in autophagy initiation, particularly under conditions of low nutrient availability (42). However, the effect of ULK1 deficiency was limited to autophagy initiation and did not seem to impact overall protein degradation because the levels of ubiquitinated proteins were unchanged. ULK1 deficiency, as assessed by 2 independent miRs, also had no apparent effect on the protein or mRNA levels of the ubiquitin and autophagy adaptors *p62* and *Nbr1* (Fig. 3*D*–*F* and Supplemental Fig. S3*C, D*).

These data indicate that ULK2 plays an important role in skeletal muscle protein homeostasis and a different one from ULK1. In addition, deficiency of either ULK2 or ULK1 did not impact phosphorylation levels of the ULK1 putative targets Atg13 (Ser318) (43) and Atg14 (Ser29) (44) but resulted in increased Atg13 protein levels, likely due to increased protein stability because *Atg13* mRNA was unchanged by these interventions (Supplemental Fig. S4*A*–*F*). Therefore, these common signaling features in ULK2- and ULK1-deficient muscles could not explain the divergent phenotypes observed.

### ULK2 protects skeletal muscle from deposition of insoluble ubiquitinated protein aggregates

**Figure 4:**
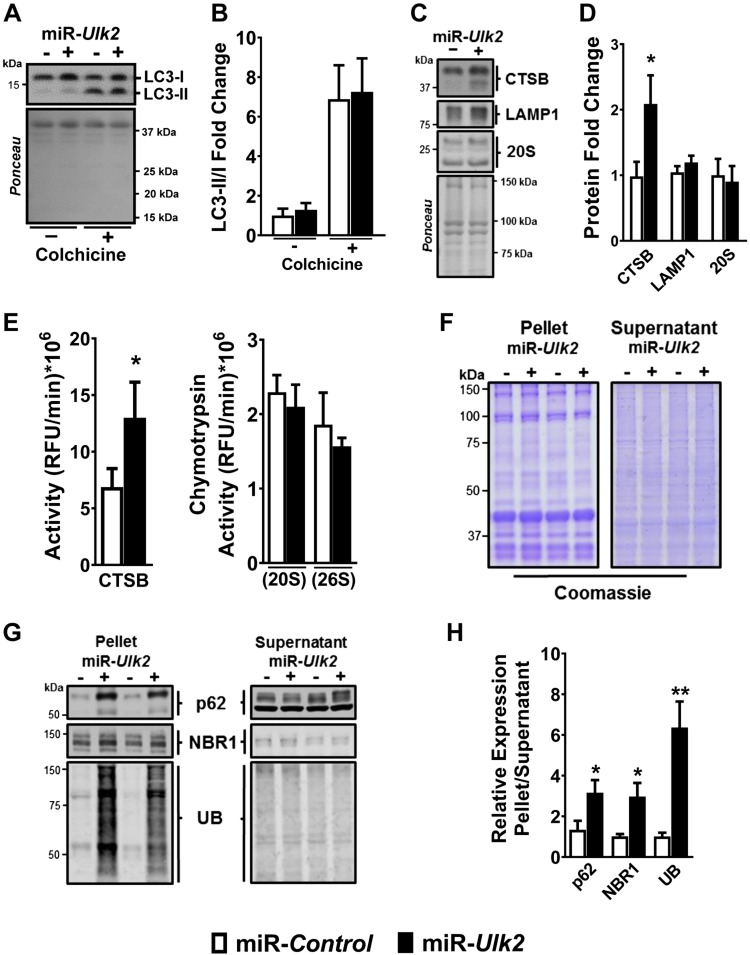
ULK2 deficiency causes deposition of insoluble ubiquitinated protein aggregates without impairing autophagy or the proteasome. Data were obtained from control and ULK-deficient TA muscles 1 wk after electroporation. *A*) Representative immunoblots of LC3-I and LC3-II in vehicle (−)– or colchicine (+)–treated control and ULK2-deficient muscles. *B*) Quantification of LC3-II/LC3-I immunoblot (*n* = 5). *C*) Representative immunoblots of CTSB, LAMP1, and 20S proteasomal subunit proteins in control and ULK2-deficient muscles. *D*) Quantification of CTSB, LAMP1, and 20S in control and ULK2-deficient muscle (*n* = 5–7). *E*) CTSB activity (left) and chymotrypsin activity (ATP-independent [20S] and ATP-dependent [26S]) (right) in control and ULK2-deficient muscles (*n* = 6). *F*) Representative Coomassie Blue stain of pellet and supernatant fractions of control and ULK2-deficient muscles. *G*) Representative immunoblots of adaptor and ubiquitinated proteins in the pellet and supernatant fractions of control and ULK2-deficient muscles. *H*) Quantification of adaptor and ubiquitinated proteins in the pellet and supernatant fractions (*n* = 7). UB, ubiquitin. Data are means ± sem. *P < 0.05, **P < 0.01.

To further explore the potential role of ULK2 in autophagy, we examined the effect of ULK2 deficiency on different aspects of the pathway. We found that ULK2 is not required for autophagosome formation or fusion to lysosome, as indicated by equal LC3-II:LC3-I ratios at baseline and upon treatment with colchicine, an inhibitor of microtubule-mediated delivery of autophagosomes to lysosomes (34, 35), in ULK2-deficient *vs.* control muscles (**Fig. 4*A, B***). In addition, ULK2 deficiency did not impair the expression or activity of lysosomal and proteasomal proteases and instead led to a potentially compensatory increase in CTSB protein and activity (Fig. 4*C*–*E*). Collectively, these findings indicate that ULK2 does not directly regulate essential steps of autophagy or the proteasome and suggest it is required for ubiquitinated protein recognition and sequestration instead. To further investigate the effect of ULK2 deficiency on ubiquitinated proteins, we used differential detergent extraction to fractionate soluble and aggregated ubiquitinated proteins from ULK2-deficient and control skeletal muscles. We found that ULK2 deficiency strongly increased the quantity of ubiquitinated protein aggregates in skeletal muscle without affecting the quantity of soluble ubiquitinated proteins (Fig. 4*F*–*H*). Thus, ULK2 plays an essential role in protein homeostasis, greatly limiting the deposition of insoluble ubiquitinated protein aggregates in skeletal muscle fibers. Nevertheless, ULK2 plays a small if any role in several critical steps of autophagy. Also, ULK2 also does not seem to directly regulate proteasome function.

### ULK2 is required for maintenance of skeletal muscle strength, mass, and myofiber integrity

**Figure 5:**
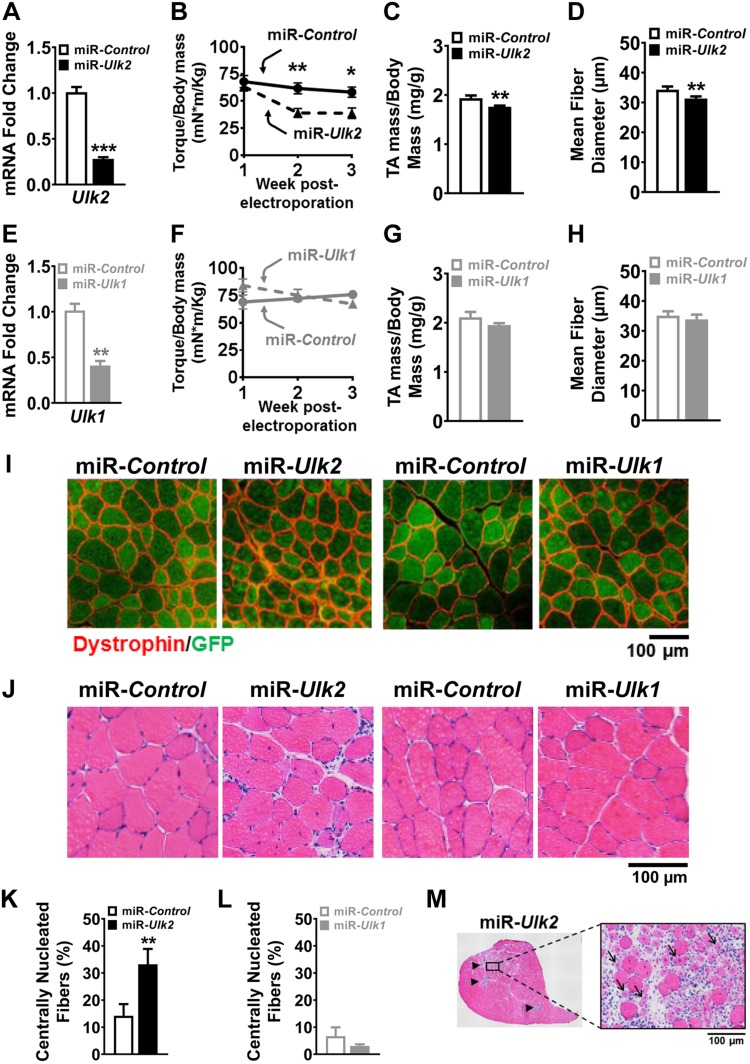
ULK2 is required for maintenance of skeletal muscle force, mass, and integrity. Data were obtained from control and ULK-deficient TA muscles 4 wk after electroporation. *A*) Relative *Ulk2* mRNA in control and ULK2-deficient muscles (*n* = 5–6). *B*) Weekly monitoring of maximal isometric torque in ankle dorsiflexors in mice with unilateral TA deficiency of ULK2 (*n* = 5). *C*) Wet muscle mass normalized to body mass in control and ULK2-deficient muscles (*n* = 5). *D*) Mean muscle fiber diameter as previously indicated in *C* (*n* = 6). *E*) Relative *Ulk1* mRNA in control and ULK1-deficient muscles (*n* = 5–6). *F*) Weekly monitoring of maximal isometric torque in ankle dorsiflexors in mice with unilateral TA deficiency of ULK1 (*n* = 6). *G*) Wet muscle mass normalized to body mass in control and ULK1-deficient muscles (*n* = 6). *H*) Mean muscle fiber diameter as previously indicated in *G* (*n* = 6). *I*) Representative images of myofibers following immunofluorescence staining for dystrophin and GFP in control and either ULK2- or ULK1-deficient muscles. *J*) Representative H&E images denoting centrally nucleated fibers in ULK2-deficient muscle. *K*) Quantification of centrally nucleated fibers in control and ULK2-deficient muscles (*n* = 6). *L*) Quantification of centrally nucleated fibers in control and ULK1-deficient muscles (*n* = 6). *M*) Representative H&E images of an entire cross section of ULK2-deficient muscle depicting areas of myofiber degeneration and regeneration (black arrow heads, left) and an enlarged area denoting several abnormally small, centrally nucleated, degenerating fibers surrounded by infiltrating cells (black arrows, right). Data are means ± sem. *P < 0.05, **P < 0.01, ***P < 0.001.

In skeletal muscle, insoluble protein aggregates accumulate with advanced age and many disease processes (12, 17, 45, 46). In addition, these insoluble protein aggregates are toxic to myofibers and thus are thought to play an important pathogenic role in skeletal muscle weakness and atrophy. These considerations led us to hypothesize that longer periods of ULK2 deficiency might reduce skeletal muscle strength, mass, and integrity. In support of this hypothesis, reduced maximal isometric torque became evident after 2 wk of ULK2 deficiency, along with decreased muscle mass at 4 wk (**Fig. 5*A*–*C***). Histologic evaluation at 4 wk of ULK2 deficiency revealed a small but significant reduction in myofiber size, a robust increase of centrally nucleated myofibers, and evidence for muscle degeneration and regeneration in discrete areas with very small and multinucleated myofibers surrounded by infiltrating cells (Fig. 5*D, I, J, K, M*). In contrast, ULK1 deficiency had no discernable effect on muscle force, mass, or morphology (Fig. 5*E*–*J, L*). Accordingly, accumulation of ubiquitinated proteins at this later time point was still evident in ULK2-deficient muscles but not in ULK1-deficient muscles (Supplemental Fig. S5*A*–*D*). Thus, ULK2 and ULK1 have dramatically different roles in skeletal muscle, with ULK1 regulating initiation but not muscle force, muscle mass, or myofiber integrity and ULK2 regulating the deposition of insoluble ubiquitinated protein aggregates, muscle force, muscle mass, and myofiber integrity.

## DISCUSSION

**Figure 6:**
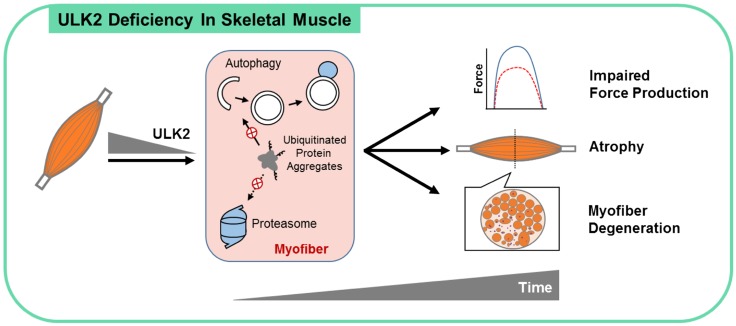
ULK2 is essential for skeletal muscle homeostasis. Our studies reveal that the *Ulk2* gene presents a skeletal muscle–enriched pattern of expression in mice and that its deficiency in skeletal muscle, despite not impairing autophagy flux and proteolytic activities of the lysosome and proteasome, leads to robust accumulation of insoluble ubiquitinated protein aggregates associated with the adaptors p62 and NBR1. These findings suggest a key role for ULK2 in modulating the recognition and sequestration of ubiquitinated protein aggregates for degradation by autophagy (and potentially by the proteasome). The ensuing inability of ULK2-deficient muscle fibers to clear proteotoxic aggregates leads to atrophy, impaired force production, myofiber degeneration, and a generally unhealthy morphology of the muscle. Of note, these cellular events and functional outcomes are not observed in ULK1-deficient muscle. Autophagy is depicted with a phagophore and an autophagosome (white) and lysosome (blue).

Several important findings arise from this study (**Fig. 6**). First, despite being expressed in several tissues, *Ulk2* is enriched in skeletal muscle. Our findings demonstrate that ULK2 is required for normal ubiquitinated protein degradation. Given the fact that a major portion of proteins in skeletal muscle is degraded upon polyubiquitination (23), a disruption of this process would likely have broad effects on tissue homeostasis. In fact, adult ULK2 deficiency in muscle causes accumulation of insoluble ubiquitinated protein aggregates and a myopathy characterized by myofiber weakness, atrophy, and degeneration. Second, despite being the closest mammalian homologs of Atg1, ULK1 and ULK2 have distinct functions in skeletal muscle. Our results are consistent with ULK1 modulating autophagy initiation while revealing a novel and unique role for ULK2 regulating ubiquitinated cargo recognition and sequestration in skeletal muscle.

Curiously, deficiency of ULK2 in skeletal muscle does not cause an overall disruption of autophagy as seen with deficiency of Atg7, in which accumulations of p62, NBR1, and ubiquitinated proteins are observed together with a major impairment in autophagy initiation (47). In fact, our results demonstrate that despite the disrupted degradation of p62, NBR1, and ubiquitinated proteins early on during ULK2 deficiency, essential components of autophagy, such as initiation (*i.e.*, LC3-I to LC3-II conversion), autophagosome fusion with lysosomes, and lysosome protease activity are preserved. Therefore, ULK2 deficiency dissociates autophagic initiation and resolution from the degradation of p62, NBR1, and ubiquitinated proteins. This indicates that skeletal muscle ULK2 modulates the recognition and sequestration of ubiquitinated cargo for proteolysis likely in a p62- and NBR1-dependent manner. Of note, p62 also serves as an adaptor for ubiquitinated protein degradation *via* the proteasome by interacting with 26S Proteasome AAA-ATPase Subunit Rpt1 [also known as PSMC2 (Proteasome 26S Subunit, ATPase 2)] (48), an ATPase integrating the 26S proteasome. Therefore, we cannot rule out a small contribution of potentially reduced proteasome-mediated protein degradation to the deposition of insoluble ubiquitinated protein aggregates observed upon ULK2 deficiency.

Previous studies in fibroblasts, neurons, astrocytes, and adipocytes have observed both redundant and different functions for ULK2 and ULK1 in the regulation of autophagy and other cellular processes (49–51). Accordingly, ULK2 and ULK1 have been found to integrate different macromolecular complexes in HEK-293A cells (52). In that sense, the contrasting functional roles of ULK2 and ULK1 observed suggest the existence of a specific interactome for each ULK also in skeletal muscle. Future studies characterizing proteins specifically interacting with (and perhaps being phosphorylated by) each ULK in skeletal muscle are granted and will likely provide important new insights into the regulation of skeletal muscle proteostasis under both physiologic and pathologic conditions.

Stimulation of autophagy by nutrient deprivation is conserved from yeast to mammals, and starvation robustly stimulates autophagy in skeletal muscle. Nevertheless, the present study demonstrates that neither ULK2 nor ULK1 is required for starvation-induced skeletal muscle atrophy. Considering that ULK1 deficiency led to a significant yet mild defect in autophagy initiation during starvation, a negligible role for ULK1 in the resulting atrophy is not surprising. In addition, the evidence that ubiquitinated proteins accumulate in ULK2-deficient muscles of mice with normal food access and that only a modest increase in accumulation is observed with starvation suggests that most of the additive degradation of ubiquitinated proteins that occurs under low nutrient availability is independent of ULK2. Given that the ubiquitinated protein accumulation seen in ULK2-deficient muscle is primarily caused by the impaired degradation of insoluble ubiquitinated protein aggregates, we can further conceive that the large majority of proteins degraded during fasting is soluble and ULK2 is indispensable for the degradation of insoluble ubiquitinated protein aggregates even when overall autophagic and proteasomal proteolytic rates are largely increased (as seen with fasting).

The precise molecular mechanisms by which ULK2 prevents the deposition of insoluble ubiquitinated protein aggregates remains to be elucidated. Our data suggest that ULK2 regulates the interaction of p62- and NBR1-linked ubiquitinated protein aggregates with proteins in the autophagosome (*i.e.*, LC3 and LC3-like proteins) and, potentially, in the proteasome (*e.g.*, Rpt1). However, this ULK2-mediated modulation is independent from and cannot be compensated by p62 Ser403 phosphorylation (Ser405 in mouse p62), which stimulates the degradation of ubiquitinated proteins (53, 54) but is actually enhanced in ULK2-deficient muscle (Supplemental Fig. S6*A, B*).

In summary, our results demonstrate an essential and unique role for ULK2 in regulating the degradation of insoluble ubiquitinated protein aggregates in skeletal muscle. Therefore, ULK2 might represent a novel therapeutic target to enhance basal selective protein degradation efficiency and turnover. It will be important to investigate this prospect in conditions associated with poor protein homeostasis in skeletal muscle as observed in metabolic diseases, several myopathies, and aging.

## Supplementary Material

This article includes supplemental data. Please visit *http://www.fasebj.org* to obtain this information.

## Abbreviations

Atg: autophagy related protein
CTSB: cathepsin B
DAVID: Database for Annotation, Visualization and Integrated Discovery
EmGFP: emerald GFP
GFP: green fluorescent protein
H&E: hematoxylin and eosin
LAMP1: lysosomal associated membrane protein 1
Lc3: light chain 3
Map1lc3: microtubule associated protein 1 Lc3
miR: microRNA
NBR1: next to breast cancer type 1 susceptibility protein gene 1 protein
qPCR: quantitative PCR
SQSTM1: sequestosome 1
TA: tibialis anterior
ULK: unc-51 like autophagy activating kinase
