# Supplementary material for: ULK2 is essential for degradation of ubiquitinated protein aggregates and homeostasis in skeletal muscle
Source: FASEB J. 2019 Aug 9;33(11):11735–45. doi: 10.1096/fj.201900766R (PMC6902739; doi:10.1096/fj.201900766R)
Supplement: Supplementary file 4 [file fj.201900766R.st4.docx]

**Supplemental Table 4:** **Primers used in qPCR analysis of mRNA expression.**

| **mRNA** | **Forward Primer (5’ to 3’)** | **Reverse Primer (3’ to 5’)** |
| --- | --- | --- |
| *Ulk1* | AACATCGTGGCGCTGTATGA | TGCGCATAGTGTGCAGGTAG |
| *Ulk2* | AATCTTGCCCAGTCCCAGTG | GGAAGGGATGGTGGAGAAGC |
| *Lc3a* | GACCGGCCTTTCAAGCAG | TGGGACCAGAAACTTGGTCT |
| *Lc3b* | CGTCCTGGACAAGACCAAGT | ACCATGTACAGGAAGCCGTC |
| *Nbr1* | CCCCAGATTGGTTTACAAGC | TCCACCGTTTCCTTAACCAC |
| *p62* | TGGGCAAGGAGGAGGCGACC | CCTCATCGCGGTAGTGCGCC |
| *Ubb* | GCGGTTTGTGCTTTCATCAC | CTCTCAGGCGAAGGACCA |
| *Ubc* | AGTCGCCCGAGGTCACAG | TCTCACGGAGTTGTTTCACG |
| *Gapdh* | AACGACCCCTTCATTGAC | TCCACGACATACTCAGCAC |
