# Supplementary material for: ULK2 is essential for degradation of ubiquitinated protein aggregates and homeostasis in skeletal muscle
Source: FASEB J. 2019 Aug 9;33(11):11735–45. doi: 10.1096/fj.201900766R (PMC6902739; doi:10.1096/fj.201900766R)
Supplement: Supplementary file 5 [file fj.201900766R.sd1.pdf]

Supplemental Figure 1

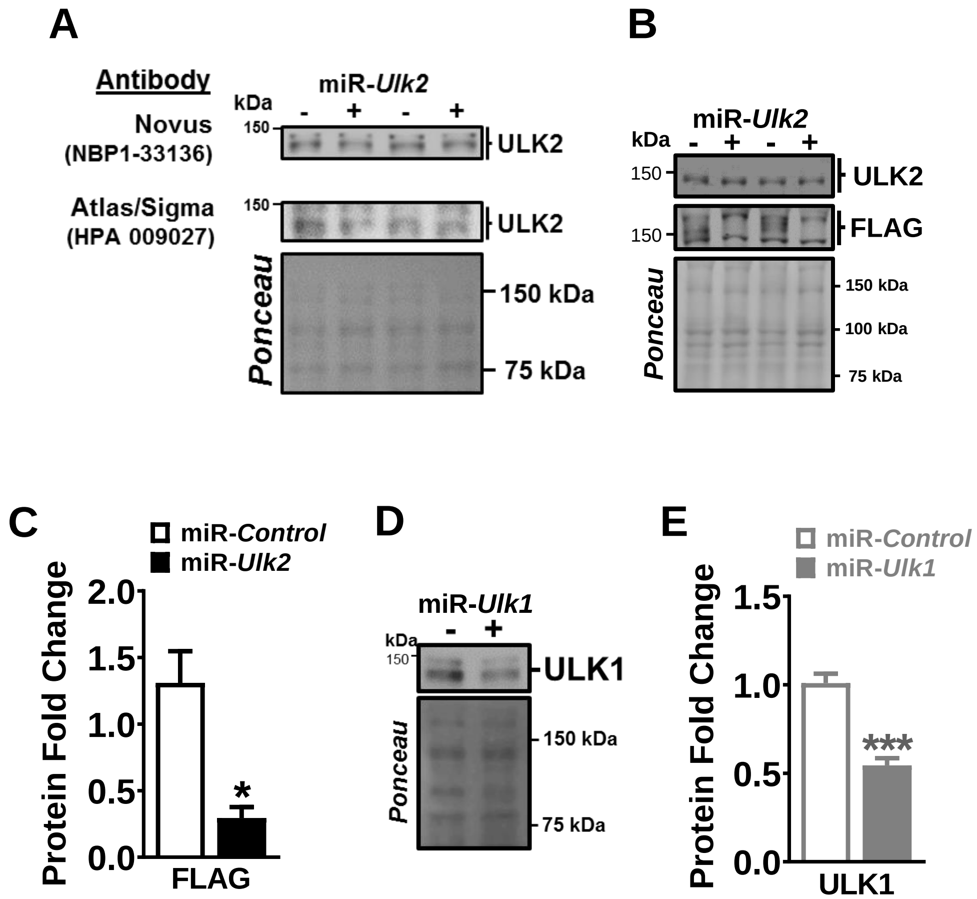

Supplemental Figure 1: Impact of miR-*Control*, miR-*Ulk2* and miR-*Ulk1* on target proteins. Data obtained from control and ULK deficient TA muscles, 1 week after electroporation. **A)** Representative Immunoblots for ULK2 using two different ULK2 primary antibodies failed to detect changes in ULK2 protein in muscles transfected with miR-*Ulk2* vs. miR-*Control*. **B)** Representative immunoblots of ULK2 and FLAG in muscles transfected with wild type [WT]-ULK2 containing N-terminal FLAG tags and miR-*Control* and contralateral muscles transfected with WT-ULK2 and miR-*Ulk2*. **C)** Quantification of FLAG expression in miR-*Control* and miR-*Ulk2* muscles (as described in B) demonstrating that miR-*Ulk2* is effective at reducing ULK2 protein (N=5). **D)** Representative immunoblot of ULK1 in muscles transfected with miR-*Ulk1* and miR-*Control*. **E)** Quantification of ULK1 as described in D (N=6). Data are means ± SEM; *P<0.05, ***P<0.001.

Supplemental Figure 2

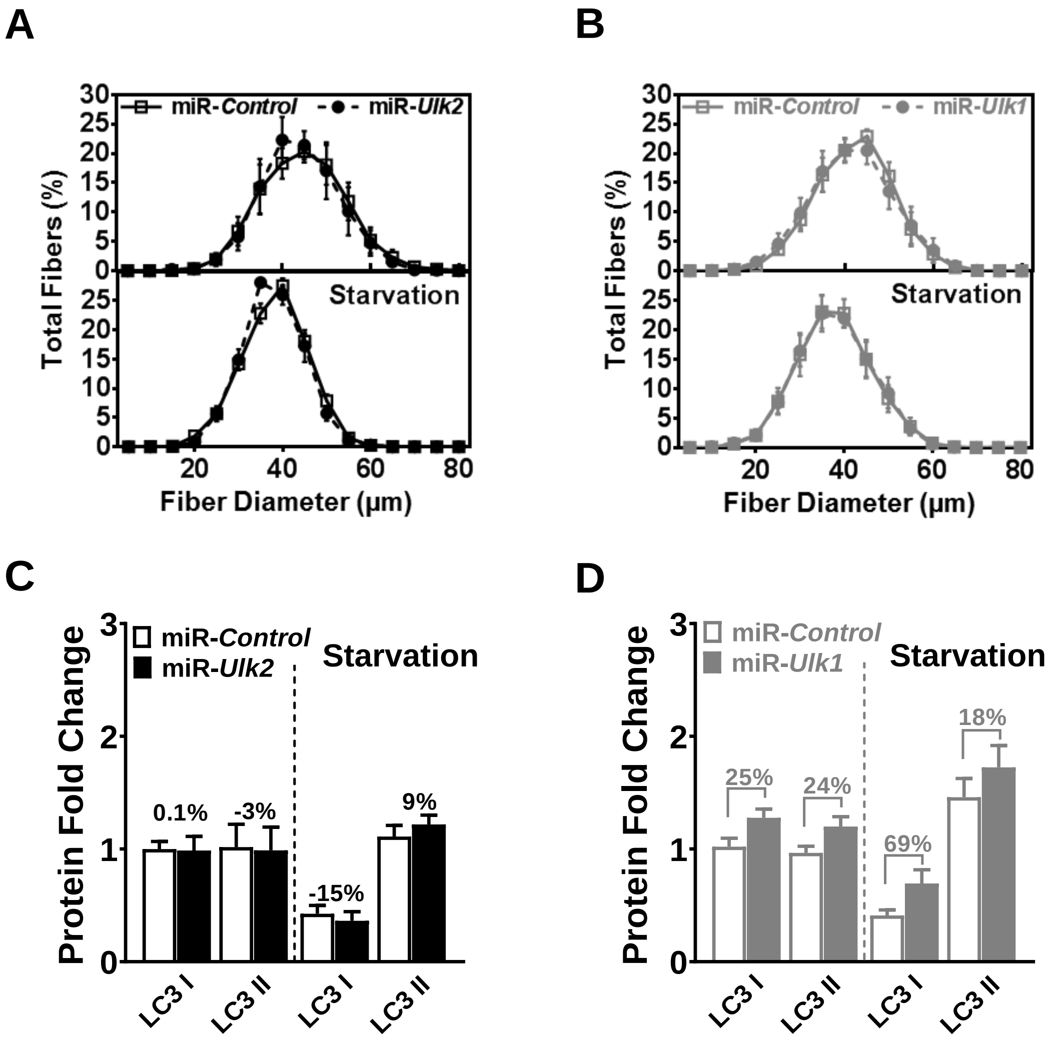

Supplemental Figure 2: Fiber diameter distribution and quantification of LC3-I and LC3-II proteins in muscles with ULK deficiency under normal or starvation conditions. Data obtained from control and ULK deficient TA muscles, 1 week after electroporation. **A)** Fiber diameter distribution in control and ULK2 deficient muscles of mice with normal food access or after 48h of starvation (N=6-9). **B)** Fiber diameter distribution in control and ULK1 deficient muscles of mice with normal food access or after 48h of starvation (N=7-8). Note: myofiber diameter distribution in control muscles under basal and starvation conditions were undistinguishable from ULK (ULK1 or ULK2) deficient muscles. **C)** Quantification of LC3-I and LC3-II levels (pertinent to data shown in Fig. 3A) in control and ULK2 deficient muscles of mice with normal food access (*left*) or after 24h of starvation (*right*) (N=7). Percent changes for each protein in relation to miR-*Control* mean are shown. **D)** Quantification of LC3-I and LC3-II levels (pertinent to data shown in Fig. 3D) in control and ULK1 deficient muscles of mice with normal food access (*left*) or after 24h of starvation (*right*) (N=6-8). Percent changes for each protein in relation to miR-*Control* mean are shown. Data are means ± SEM.

Supplemental Figure 3

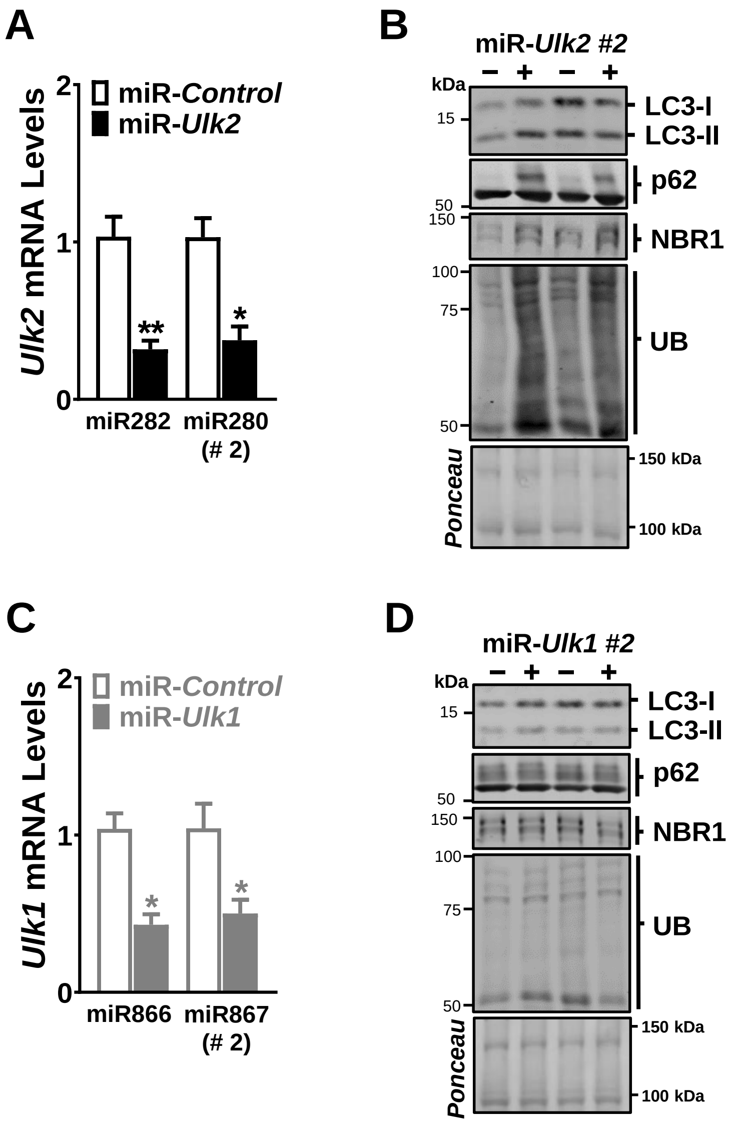

Supplemental Figure 3: Impact of a second independent miRNA targeting either *Ulk2* or *Ulk1* on autophagy markers in skeletal muscle. Data obtained from control and ULK deficient TA muscles in mice with normal food access, 1 week after electroporation. **A)** Relative *Ulk2* mRNA in control and ULK2 deficient muscle electroporated with either Mmi541282 (miR282 – referred to as miR-*Ulk2*) and Mmi541280 (miR280 – referred to as miR-*Ulk2* #2). Both miRs led to 50% or greater reduction in *Ulk2* mRNA (N=5). **B)** Representative immunoblots of LC3, adaptor proteins, and ubiquitinated proteins in control and ULK2 deficient muscles electroporated with miR-*Ulk2* #2. Results recapitulate findings observed with miR-*Ulk2* shown in Fig. 3A. **C)** Relative *Ulk1* mRNA in control and ULK1 deficient muscles electroporated with either Mmi525866 (miR866 – referred to as miR-*Ulk1*) and Mmi525867 (miR867 – referred to as miR-*Ulk1* #2). Both miRs led to 50% or greater reduction in *Ulk1* mRNA (N=5). **D)** Representative immunoblots of LC3, adaptor proteins, and ubiquitinated proteins in control and ULK1 deficient muscles electroporated with miR-*Ulk1* #2. Results recapitulate findings observed with miR-*Ulk1* shown in Fig. 3D. Data are means ± SEM; *P<0.05, **P<0.01.

Supplemental Figure 4

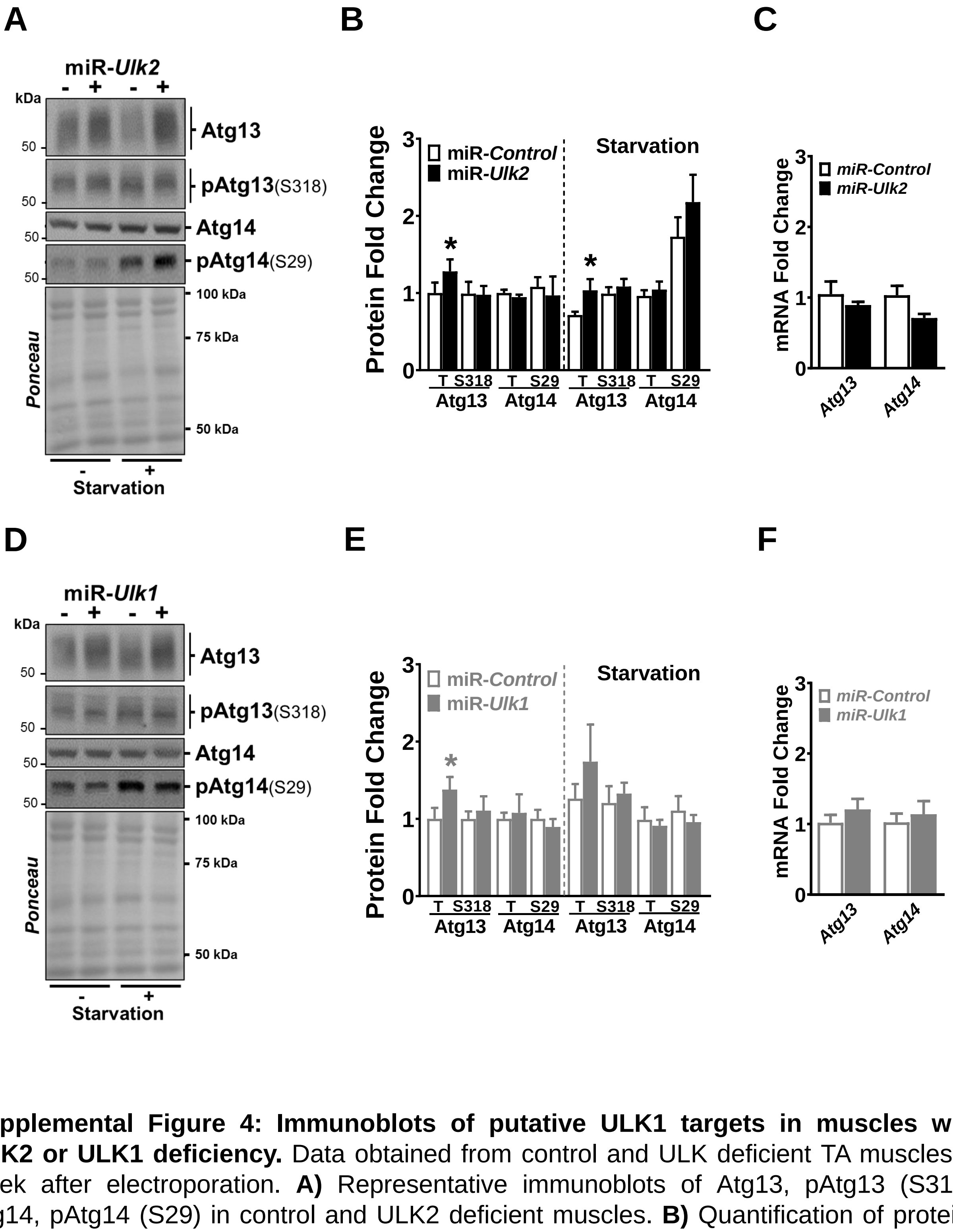

Supplemental Figure 4: Immunoblots of putative ULK1 targets in muscles with ULK2 or ULK1 deficiency. Data obtained from control and ULK deficient TA muscles, 1 week after electroporation. **A)** Representative immunoblots of Atg13, pAtg13 (S318), Atg14, pAtg14 (S29) in control and ULK2 deficient muscles. **B)** Quantification of proteins shown in A (N=7-8). **C)** Relative *Atg13* and *Atg14* mRNA in control and ULK2 deficient muscle (N=5) **D)** Representative immunoblots of Atg13, pAtg13 (S318), Atg14, pAtg14 (S29) in control and ULK1 deficient muscles. **E)** Quantification of proteins shown in C (N=5-8). **F)** Relative *Atg13* and *Atg14* mRNA in control and ULK1 deficient muscle (N=4). Data are means ± SEM; *P<0.05.

Supplemental Figure 5

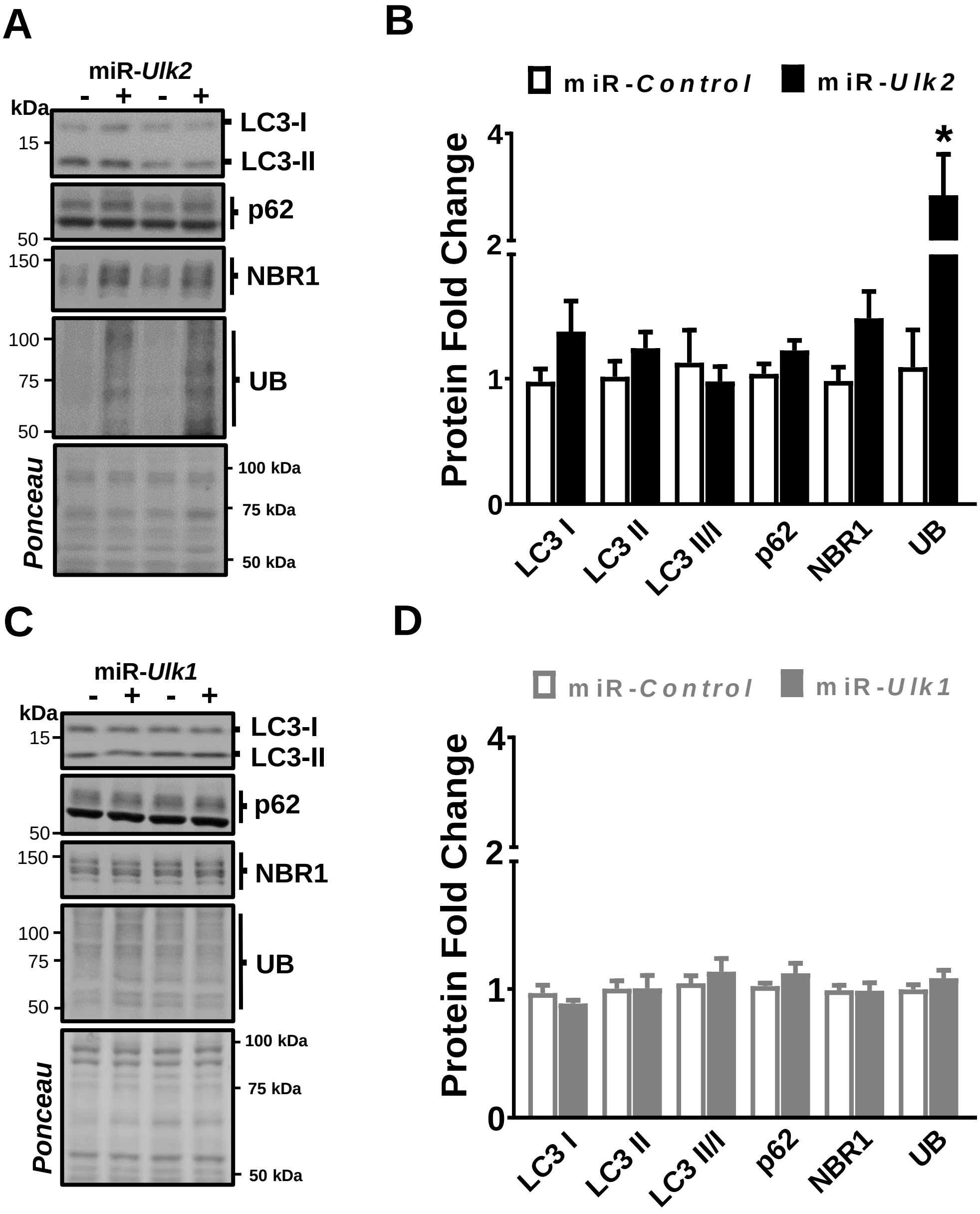

Supplemental Figure 5: Quantification of LC3, protein adaptors and ubiquitinated proteins after 4 weeks of ULK2 or ULK1 deficiency in skeletal muscle. Data obtained from control and ULK deficient TA muscles, 4 weeks after electroporation. **A)** Representative immunoblots of LC3, autophagy adaptors (p62 and NBR1), and ubiquitinated proteins in control and ULK2 deficient muscles. **B)** Quantification of proteins shown in A (N=5). **C)** Representative immunoblots of LC3, autophagy adaptors (p62 and NBR1), and ubiquitinated proteins in control and ULK1 deficient muscles. **D)** Quantification of proteins shown in C (N=6). Data are means ± SEM; *P<0.05.

Supplemental Figure 6

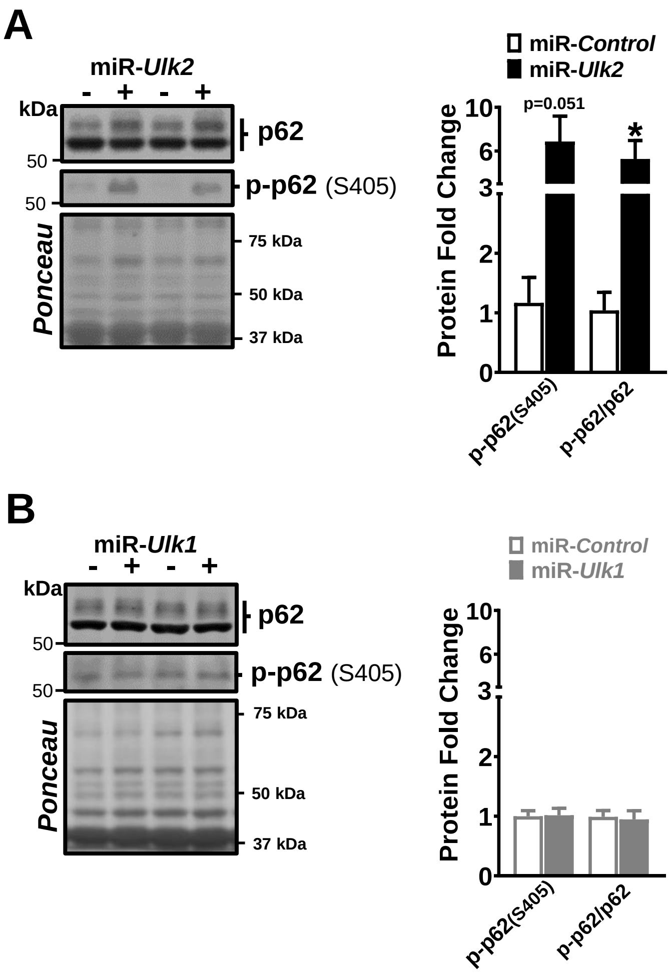

Supplemental Figure 6: Immunoblots of p62 and p-p62 (Ser 405) after 4 weeks of ULK2 or ULK1 deficiency. Data obtained from control and ULK deficient TA muscles, 4 weeks after electroporation. **A)** Representative immunoblots and quantification of p62 and p-p62 (S405) in control and ULK2 deficient muscles (N=5). **B)** Representative immunoblots and quantification of p62 and p-p62 (S405) in control and ULK1 deficient muscles (N=6). Data are means ± SEM; *P<0.05. Exact P values denoting statistical trends (P<0.1) are shown when applicable.
